# Long-term postoperative outcomes of Roux-en-Y cholangiojejunostomy in patients with benign biliary stricture

**DOI:** 10.1186/s12893-022-01622-y

**Published:** 2022-06-16

**Authors:** Paizula Shalayiadang, Aimaiti Yasen, Abduaini Abulizi, Ayifuhan Ahan, Tiemin Jiang, Bo Ran, Ruiqing Zhang, Qiang Guo, Hao Wen, Yingmei Shao, Tuerganaili Aji

**Affiliations:** 1 Department of Hepatobiliary and Echinococcosis Surgery, Digestive and Vascular Surgery Center, The First Affiliated Hospital of Xinjiang Medical University, Urumqi, 830054 Xinjiang China; 2 Department of Hepatobiliary Surgery, The First Affiliated Hospital of Guangzhou Medical University, Guangzhou, 510120 Guangdong China; 3 State Key Laboratory of Pathogenesis, Prevention and Management of High Incidence Diseases in Central Asia, First Affiliated Hospital of Xinjiang Medical University, Urumqi, 830054 Xinjiang China

**Keywords:** Biliary stricture, Cholangiojejunostomy, Outcomes, Complications, Survival

## Abstract

**Background:**

Although there are common postoperative complications, Roux-en-Y cholangiojejunostomy is still broadly used as a standard surgical procedure for patients with biliary stricture. This study aimed to explore long-term risk factors of cholangiojejunostomy in patients with biliary stricture who underwent revisional cholangiojejunostomy.

**Methods:**

Clinical data of 61 patients with biliary stricture undergoing revisional cholangiojejunostomy were retrospectively analyzed. These patients were classified into two groups (patients with traumatic biliary stricture and non-traumatic biliary stricture). Postoperative complications and survival time were successfully followed up.

**Results:**

Among the patients, 34 underwent revisional cholangiojejunostomy due to traumatic biliary stricture, and 27 underwent revisional cholangiojejunostomy due to non-traumatic biliary surgery. Although there was no statistical difference in most clinical data between two groups, biliary dilation or not during the first surgery, cholelithiasis or not during the first surgery, long-term complications after first surgery, cholelithiasis or not during the second surgery, identifying abnormalities during the second surgery and long-term complications after second surgery were significantly different. All patients were successfully followed up and average follow-up time for patients with traumatic and non-traumatic biliary stricture was (88.44 ± 35.67) months and (69.48 ± 36.61) months respectively. Survival analysis indicated that there was no statistical difference in overall survival between two groups. Additionally, cox proportional hazard analysis demonstrated that first preoperative bilirubin level, short-term complication after first surgery and identifying abnormalities during the second surgery were independent risk factors that may have significant effects on patients' overall survival and long-term prognosis after cholangiojejunostomy. Among the intraoperative abnormal findings, residual lesions after the first operation had significant effects on the patients overall survival in the earlier stage. Relatively, anastomotic stoma stricture and biliary output loop problems had obvious effects on patients' overall survival at later stages.

**Conclusion:**

First preoperative bilirubin level, short-term complication after first surgery and abnormal findings during the second surgery were independent risk factors of revisional cholangiojejunostomy, which may affect patients' long-term survival. Therefore, surgeons should minimize incidence of postoperative complications through fully evaluating optimal operative time and standardizing surgical procedures.

## Background

Roux-en-Y cholangiojejunostomy, as a standard surgical procedure, is widely applied for the treatment of biliary stricture in clinical entity [1–3]. However, serious short- and long-term complications in some patients after surgery may still occur, especially postoperative biliary stricture or intrahepatic calculosis, which often need to be further treated via second or even multiple surgery, thus, causing great trauma and economic burden to the patients [4–6]. Therefore, it is of great clinical significance to select optimal treatment procedures to reduce occurrence of such potential complications.

Some significant factors must be considered in the evaluation process of long-term outcomes after Roux-en-Y cholangiojejunostomy. Most importantly, it is crucial to unify evaluation measures of long-term outcomes in specific clinical scenario, which should include the following aspects: (i) clinical manifestations and physical signs of the patients; (ii) assessing methods of patients' liver functions; (iii) representative imaging. In addition, follow-up time period was another imperative element that should be taken into consideration when analyzing long-term prognosis of patients who underwent cholangiojejunostomy [7–10]. It has been shown by Tochhi et al. that approximately 40% of recurrent biliary strictures were diagnosed 5 years after the initial surgery [11]. Therefore, comprehensive and accurate evaluation of long-term outcomes of Roux-en-Y cholangiojejunostomy may need at least 5-year or longer follow-up period. Numerous clinical studies have demonstrated that a number of key factors, including patients' preoperative physical condition, different anatomotic methods, lesion sites and postoperative complications, may have potential effects on patients' prognosis and overall study after Roux-en-Y cholangiojejunostomy [12–14]. Herein, this study was aimed to provide reference for the improvement of such patients' prognosis through analyzing possible risk factors of Roux-en-Y cholangiojejunostomy in 61 patients who underwent this surgical procedure for the second time.

## Methods

### Study subjects

A retrospective case–control study involving 61 patients who underwent revisional Roux-en-Y cholangiojejunostomy at our department were conducted in this study. Among the patients, 34 underwent revisional Roux-en-Y cholangiojejunostomy due to traumatic biliary stricture, and the rest 27 patients underwent revisional cholangiojejunostomy because of non-traumatic biliary stricture mainly caused by chronic cholangitis. Study subjects were selected according to the following indications: (i) Diagnosis of biliary stricture was clearly determined through preoperative imaging and postoperative histological findings of surgical specimens. (ii) Patients underwent Roux-en-Y cholangiojejunostomy due to biliary stricture at the first time, and there was anastomotic stoma stricture or intrahepatic biliary duct calculosis postoperatively during the follow-up and then received revisional cholangiojejunostomy. (iii) Patients were no accompanied with severe cardiopulmonary dysfunction and and could tolerate open surgery. (iv) Postoperative clinical data of the patients were successfully followed up.

### Preoperative assessment

**Fig. 1:**
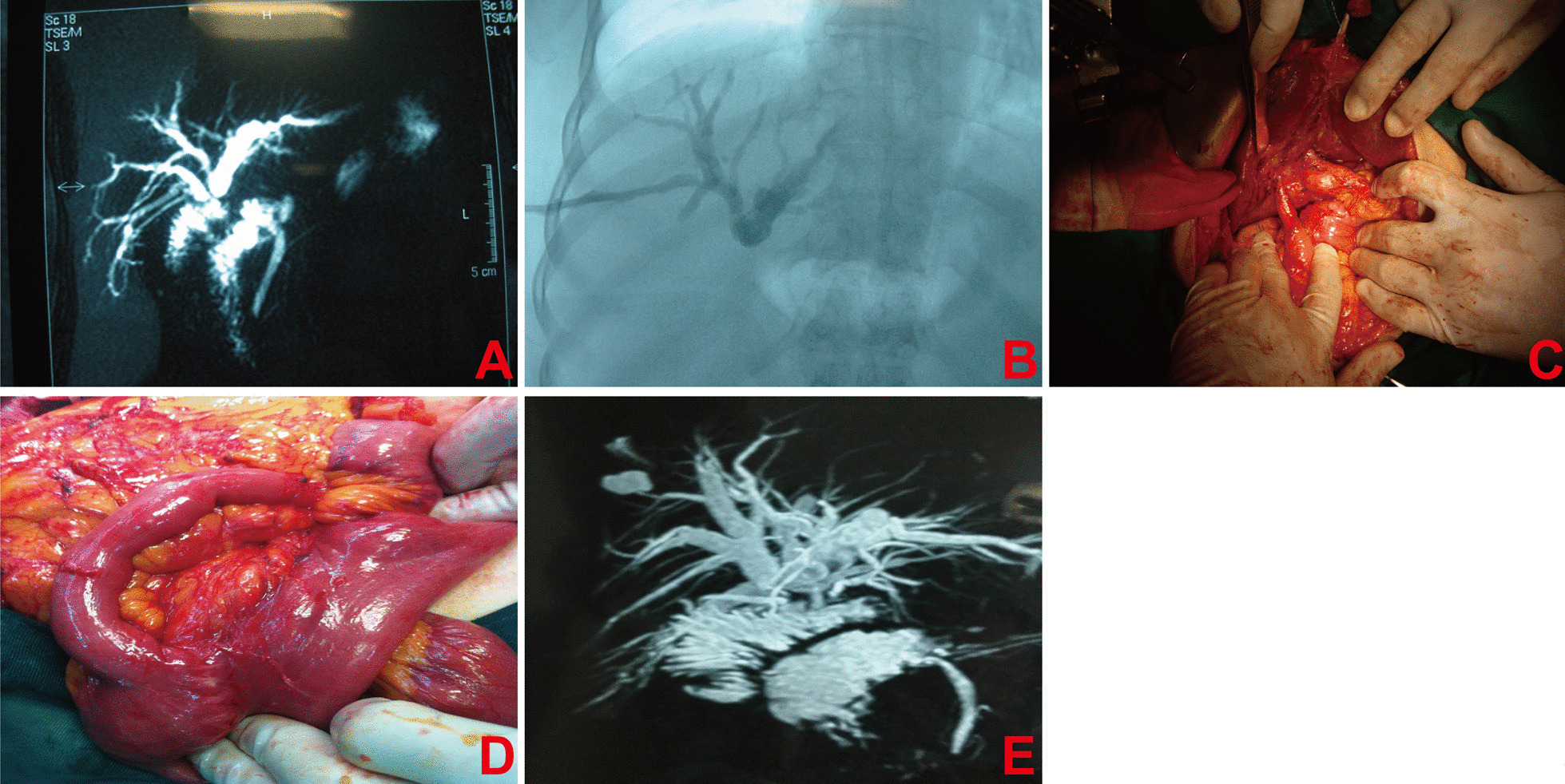
Typical imaging and intraoperative photos of the patients with traumatic biliary stricture. **A** Preoperative transhepatic cholangiography showed that there formed a common trunk between the right anterior bile duct confluence and the left hepatic duct, and there was obvious biliary stricture in the left hepatic biliary orifice. **B** Radiography indicated that there was no anastomotic stoma, suggesting that the common trunk between the right anterior bile duct and the left hepatic duct was not anatomized. **C** Intraoperative imaging demonstrated obstruction of duct-jejunum anastomotic stoma and shortening of intestine in the biliary output loop. **D** Intraoperative imaging showed the significant difference between biliary output loop and the normal intestine. **E** Postoperative transhepatic cholangiography indicated obstruction of anastomotic stoma and intrahepatic biliary duct calculosis after cholangiojejunostomy

**Fig. 2:**
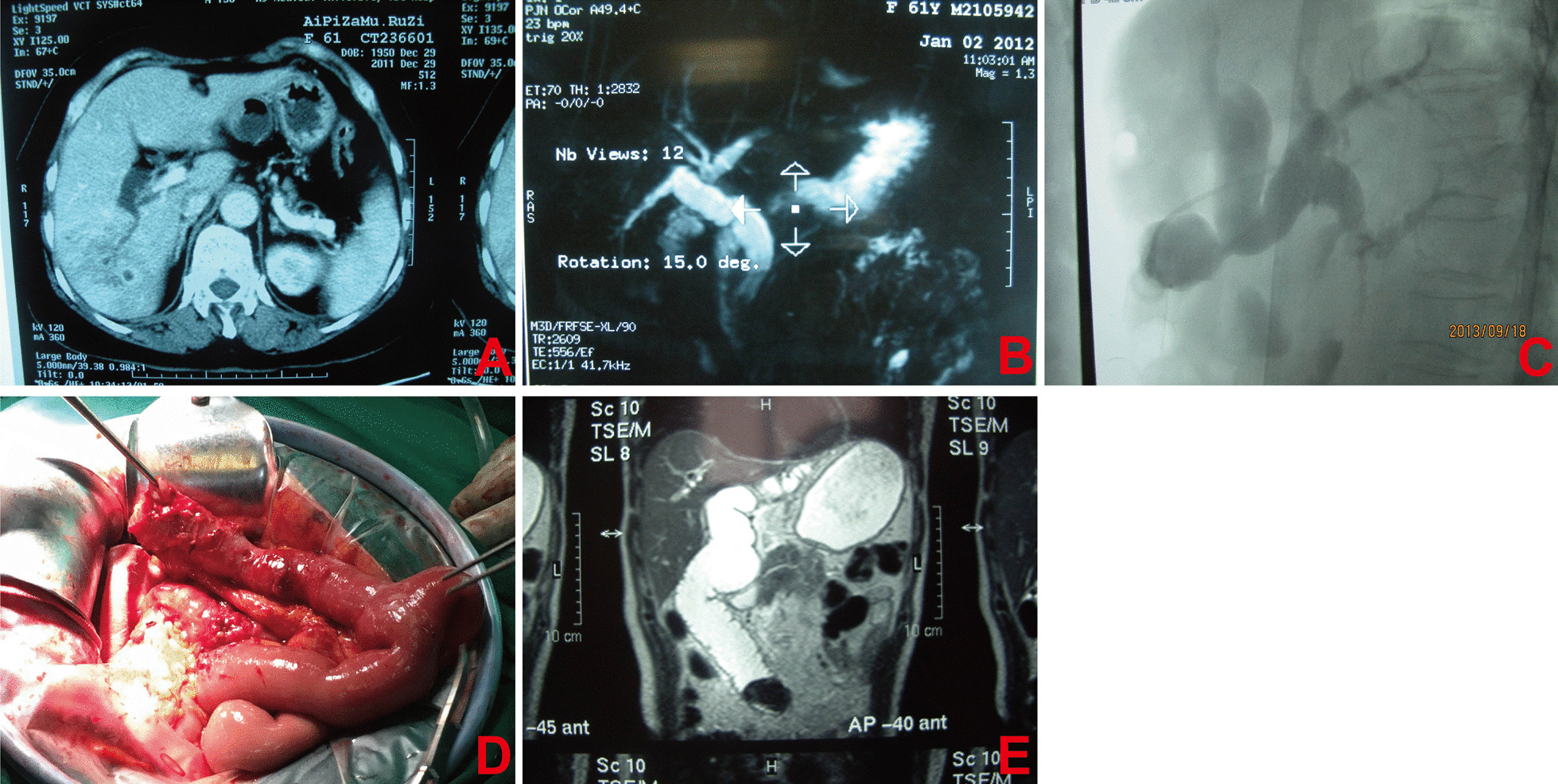
Representative imaging and intraoperative pictures of the patients with non-traumatic biliary stricture. **A** Enhanced computed tomography showed that the low confluence was located in the independent right posterior biliary of the common biliary duct, and there formed an abscess. **B** Preoperative transhepatic cholangiography indicated that the low confluence located in the independent right posterior biliary of the common biliary duct was not anatomized, and there formed calculus in the lower end of the common biliary duct. **C** Radiography demonstrated intestinal obstruction of biliary output loop in the near anastomotic stoma. **D** Intraoperative imaging showed the over-short biliary output loop. **E** Postoperative magnetic resonance imaging indicated calculous obstruction of biliary output loop after cholangiojejunostomy

Preoperative enhanced computed tomography (CT), magnetic resonance imaging (MRI), transhepatic cholangiography and radiography were performed to make preoperative diagnosis and biliary stricture degree. According to the etiology, patients were classified into traumatic and non-traumatic biliary stricture group. Representative imaging for the two group patients were shown in Figs. 1 and 2. Preoperative percutaneous transhepatic biliary drainage (PTBD) was performed for two patients, whose total bilirubin levels were higher than 129.1 μmol/L.

### Postoperative follow-up

Long-term follow-up was conducted for the patients, and abdominal CT and liver functions were reviewed in order to evaluate postoperative complications. Postoperative complications were evaluated based on the Clavein–Dindo classification system [15]. The patients were followed up from January 2005 and the deadline was January 2021 or death time of the patients.

### Statistical analysis

Statistical analysis was performed using SPSS version 21.0 (SPSS Inc, Chicago, IL, USA) and GraphPad Prism 8.0 (GraphPad Software, San Diego, CA). All quantitative data were presented as the mean ± standard deviation. Long-term risk factors were evaluated using univariate and multivariate Cox proportional hazard analysis. Statistical significance was set at the 5% level and *P* < 0.05 was considered statistically significant.

## Results

### Clinical characteristics of the patients

Among the patients, 34 underwent revisional cholangiojejunostomy due to traumatic biliary stricture and 27 underwent revisional cholangiojejunostomy because of non-traumatic biliary stricture mainly caused by chronic cholangitis. There were 23 male and 38 female patients, whose age ranged from 21 to 74 years with the mean age of (49.87 ± 12.47) years. Twenty patients received revisional cholangiojejunostomy due to biliary injuries at the first time, and the other 14 patients received this surgery due to traumatic biliary stricture. Relatively, there occurred cholangitis stenosis in 27 patients and then they underwent revisional cholangiojejunostomy, among which 21 patients had cholangitis stenosis in the lower end of common biliary duct, three had chronic pancreatitis, which further resulted in cholangitis stenosis. In addition, one patient underwent revisional cholangiojejunostomy due to Mirzzi syndrome and two patients received this surgical procedure due to hilar cholangitis stenosis.

Comparing clinical data (Table 1), there was no statistical difference in sex, age, symptoms before the first cholangiojejunostomy, first preoperative bilirubin level, first preoperative alanine aminotransferase (ALT) level, first preoperative aspartate aminotransferase (AST) level, first preoperative albumin level, first preoperative white blood cell (WBC) counts, first preoperative acute stage or not, first biliary-enteric anastomosis level, undergoing hepatectomy or not during the first operation, short-term complication after first surgery, the earliest time of cholangitis after the first surgery, the average time interval between two operations, symptoms before the second surgery, second preoperative bilirubin level, second preoperative ALT level, second preoperative AST level, second preoperative albumin level, second preoperative WBC counts, biliary dilation or not during the second surgery, second biliary-enteric anastomosis level, undergoing hepatectomy or not during the second operation and short-term complication after second surgery between two groups (*P* > 0.05). Whereas, there were significant differences in biliary dilation or not during the first surgery, cholelithiasis or not during the first surgery, long-term complications after first surgery, cholelithiasis or not during the second surgery, identifying abnormalities during the second surgery and long-term complications after second surgery between two groups (*P* < 0.05).

**Table 1:** Demographic characteristics and clinical data of the study subjects

Variance	Traumatic biliary stricture (n = 34)	Non-traumatic biliary stricture (n = 27)	*t/c*^*2*^ value	*P* value
Sex (Male:Female)	12:22	11:16	0.190	0.791
Age (year)	47.74 ± 9.65	52.56 ± 15.07	1.516	0.135
Symptoms (1)				
Abdominal pain	17	15	2.034	0.362
Jaundice	15	6		
Fever	2	6		
Biliary dilation (1)	17	25	12.730	0.001
Biliary stone (1)	1	14	19.415	0.000
Acute stage (1)	3	3	0.089	1.000
Anastomotic level (1)				
Porta hepatis anastomosis	1	3	1.639	0.313
Extrahepatic anastomosis	33	24		
Early complications (1)				
Biliary fistula	4	2	1.550	0.461
Intestinal obstruction	0	1		
Late complications (1)				
Hepatic abscess	2	7	9.694	0.008
Intestinal obstruction	0	3		
Cholangitis time (month)	40.15 ± 34.65	51.44 ± 39.14	1.194	0.237
Interval time (month)	65.68 ± 50.47	60.33 ± 41.04	0.445	0.658
Symptoms (2)				
Abdominal pain	1	1	2.107	0.349
Jaundice	12	5		
Fever	21	21		
Biliary dilation (2)	33	27	0.807	1.000
Biliary stone (2)	21	24	5.722	0.021
Anastomotic level (2)				
Porta hepatis anastomosis	24	22	2.652	0.266
Extrahepatic anastomosis	3	0		
Intrahepatic anastomosis	7	5		
Early complications (2)				
Biliary fistula	9	4		
Incisional infection	2	2		
Intestinal obstruction	0	1		
Anastomotic fistula	0	1	5.289	0.507
Gastroparesis	1	0		
Gastrointestinal bleeding	1	0		
Late complications (2)				
Death	0	3		
Anastomotic obstruction	4	0	9.816	0.044
Incisional hernia	2	0		
Intrahepatic calculosis	0	1		
Intraoperative findings (2)				
Anastomotic stenosis	28	14		
Shortening of biliary output loop	5	11	6.533	0.038
Legacy	1	2		
Hepatectomy (1)	0	1	1.280	0.443
Hepatectomy (2)	15	6	3.196	0.105
Bilirubin (1)	75.63 ± 115.90	106.90 ± 111.49	1.064	0.292
ALT (1)	131.21 ± 150.79	154.96 ± 109.32	0.687	0.495
AST (1)	140.60 ± 160.47	204.17 ± 152.69	1.570	0.122
Albumin (1)	46.48 ± 66.10	35.11 ± 3.15	0.892	0.376
WBC (1)	8.17 ± 8.39	6.53 ± 1.45	1.006	0.319
Bilirubin (2)	46.71 ± 38.45	54.24 ± 56.44	0.619	0.539
ALT (2)	136.92 ± 119.02	110.36 ± 82.87	0.985	0.329
AST (2)	141.57 ± 97.25	134.67 ± 108.24	0.262	0.794
Albumin (2)	34.79 ± 5.16	35.42 ± 4.06	0.517	0.607
WBC (2)	7.92 ± 2.48	8.49 ± 5.56	0.535	0.595
Follow-up time (month)	88.44 ± 35.67	69.48 ± 36.61	2.032	0.047

### Postoperative follow-up data

**Fig. 3:**
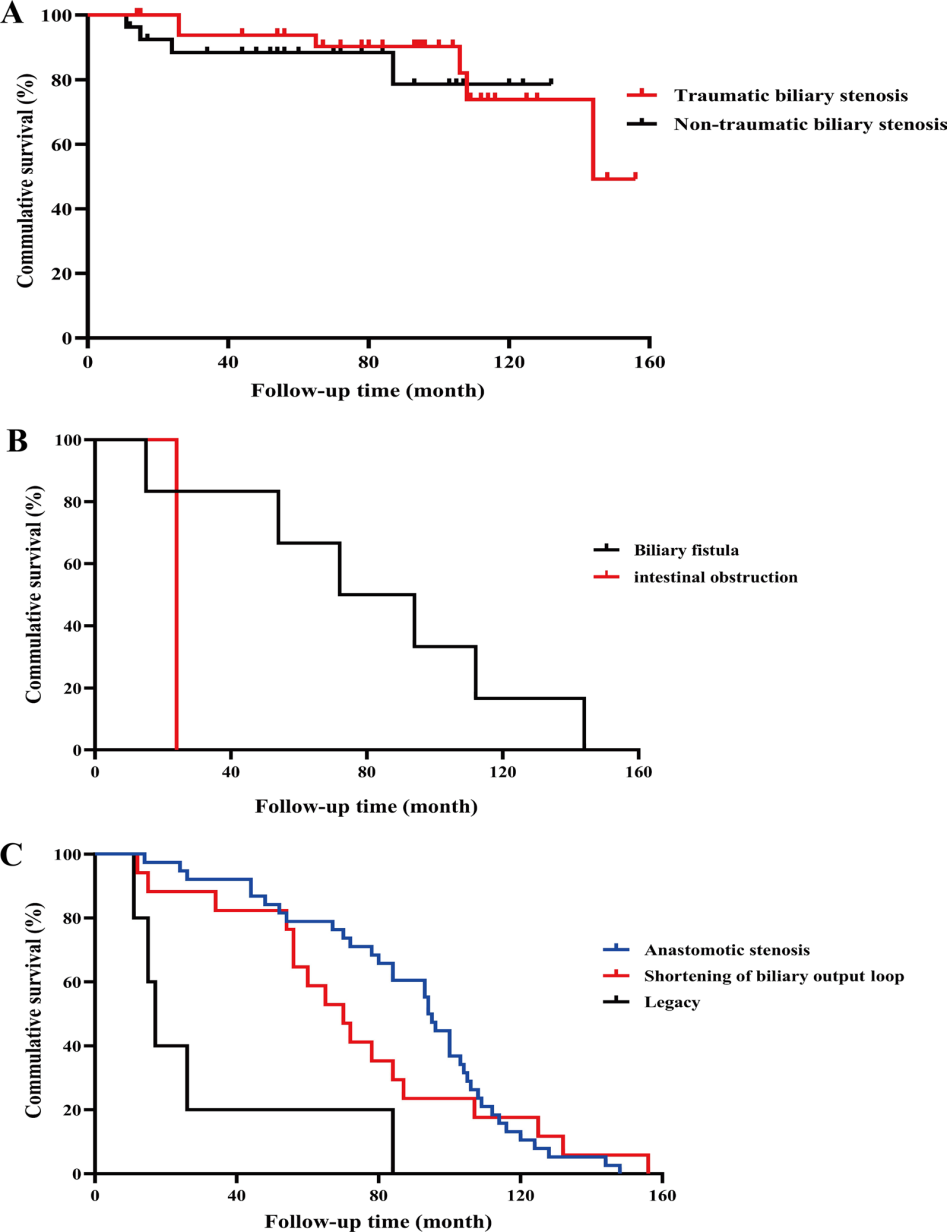
Risk factors for long-term complications of bile duct stricture after revisional cholangiojejunostomy. **A** Risks of different group patients for long-term complications of bile duct stricture after revisional cholangiojejunostomy. **B** Risks of first early complication for long-term complications of bile duct stricture after revisional cholangiojejunostomy. **C** Risks of second intraoperative findings for long-term complications of bile duct stricture after revisional cholangiojejunostomy

All patients involved in the study were successfully followed-up. The follow-up time ranged from 11 to 156 months, and average follow-up time for patients with traumatic biliary stricture was (88.44 ± 35.67) months, which was (69.48 ± 36.61) months for patients with non-traumatic stricture. During follow-up, Clavien–Dindo III and higher complications occurred in ten patients (16.4%, 10/61), including four patients with non-traumatic stricture (14.8%, 4/27) and six patients with traumatic biliary stricture (17.7%, 6/34). Three patients with non-traumatic stricture died, among whom two patients died of multiple organ failure and one died of cholangiocarcinoma. Additionally, intrahepatic biliary duct calculosis occurred in another patient. Relatively, obstruction of duct-jejunum anastomotic stoma occurred in four patients with traumatic biliary stricture and incisional hernia was also observed in another two patients. There was significant diversity in the long-term complications after surgery between two groups (*P* < 0.05. However, statistical difference in overall survival between two groups was not statistically significant (*P* > 0.05, Fig. 3A).

### Risk factors that may affect patients' prognosis after cholangiojejunostomy

In order to analysis possible risk factors of cholangiojejunostomy in patients' prognosis, univariate Cox proportional hazard analysis was used. The results demonstrated that first preoperative bilirubin level, first preoperative albumin level, first biliary-enteric anastomosis level, undergoing hepatectomy or not during the first operation, short-term complication after first surgery, second preoperative bilirubin level, biliary dilation or not during the second surgery and identifying abnormalities during the second surgery were possible risk factors that may affect patients' overall survival and prognosis after this surgery (*P* < 0.05, Table 2).

**Table 2:** Univariate Cox regression analysis of risk factors for long-term complications of biliary stricture after double choledochojejunostomy

Variance	Β value	SE	RR value	95%CI	*P* value
Different group	0.293	0.676	1.340	0.357 ~ 5.039	0.665
Sex	0.971	0.651	2.642	0.738 ~ 9.458	0.135
Age	0.002	0.027	1.002	0.951 ~ 1.055	0.944
Symptoms (1)	− 0.167	0.501	0.846	0.317 ~ 2.261	0.740
Biliary dilation (1)	− 0.167	0.650	0.846	0.237 ~ 3.028	0.798
Biliary stone (1)	− 0.025	0.803	0.975	0.202 ~ 4.707	0.975
Acute stage (1)	0.258	1.062	1.294	0.161 ~ 10.370	0.808
Anastomotic level (1)	− 2.264	0.845	0.104	0.020 ~ 0.544	0.007
Early complications (1)	1.248	0.568	3.482	1.143 ~ 10.605	0.028
Late complications (1)	0.719	0.747	2.053	0.811 ~ 5.194	0.129
Cholangitis time	− 0.013	0.010	0.987	0.968 ~ 1.006	0.170
Interval between double choledochojejunostomy	− 0.012	0.008	0.988	0.973 ~ 1.003	0.109
Symptoms (2)	1.366	1.051	3.920	0.500 ~ 30.761	0.194
Biliary dilation (2)	− 2.349	1.097	0.095	0.011 ~ 0.819	0.032
Biliary stone (2)	− 0.558	0.647	0.572	0.161 ~ 2.033	0.388
Anastomotic level (2)	0.251	0.393	1.258	0.595 ~ 2.774	0.523
Early complications (2)	− 0.249	0.350	0.506	0.393 ~ 1.548	0.477
Intraoperative findings (2)	0.928	0.424	2.529	1.102 ~ 5.803	0.029
Anastomotic stenosis	− 0.054	0.695	0.947	0.243 ~ 3.695	0.938
Hepatectomy (1)	4.069	1.414	58.484	3.657 ~ 935.175	0.004
Hepatectomy (2)	0.834	0.654	2.302	0.639 ~ 8.291	0.202
Bilirubin (1)	0.005	0.002	1.005	1.001 ~ 1.009	0.013
ALT (1)	0.000	0.003	1.000	0.995 ~ 1.005	0.938
AST (1)	0.000	0.002	1.000	0.996 ~ 1.004	0.858
Albumin (1)	− 0.191	0.098	0.826	0.682 ~ 1.001	0.051
WBC (1)	0.050	0.056	1.051	0.942 ~ 1.172	0.373
Bilirubin (2)	0.011	0.006	1.011	1.000 ~ 1.022	0.055
ALT (2)	− 0.002	0.003	0.998	0.992 ~ 1.003	0.443
AST (2)	0.001	0.003	1.001	0.998 ~ 1.007	0.845
Albumin (2)	− 0.071	0.057	0.931	0.833 ~ 1.041	0.211
WBC (2)	0.039	0.067	1.040	0.912 ~ 1.186	0.558

Risk factors that had significant difference in univariate analysis were further assessed through multivariate analysis, which indicated that first preoperative bilirubin level, short-term complication after first surgery and identifying abnormalities during the second surgery were independent risk factors that may have significant effects on patients' overall survival and long-term prognosis after the surgery (*P* < 0.05, Table 3). Further analysis confirmed that short-term complications after the first surgery, especially biliary fistula, were independent factors affecting patients' long-term survival and prognosis (Fig. 3B). Moreover, intraoperative abnormal findings during the second surgery were also significant elements that play crucial roles in the overall survival of the patients, among which residual lesions after the first operation had significant effects on patients' overall survival and may be found in the earlier stage. Relatively, anastomotic stoma stricture and biliary output loop problems had obvious effects on patients' overall survival and prognosis after surgery at later stages (Fig. 3C).

**Table 3:** Multivariate Cox regression analysis

Variance	Β value	SE	RR value	95%CI	*P* value
Anastomotic level (1)	0.936	2.294	2.550	0.028 ~ 228.405	0.683
Early complications (1)	2.485	1.183	12.003	1.181 ~ 121.960	0.036
Biliary dilation (2)	− 0.819	1.530	0.441	0.022 ~ 8.850	0.593
Intraoperative findings (2)	2.332	1.013	10.294	1.412 ~ 75.032	0.021
Hepatectomy (1)	− 3.126	2.770	0.044	0.000 ~ 10.000	0.259
Bilirubin (1)	0.010	0.005	1.010	1.000 ~ 1.019	0.049
Albumin (1)	− 0.175	0.195	0.839	0.573 ~ 1.230	3.69
Bilirubin (2)	0.010	0.013	1.010	0.984 ~ 1.037	0.436

## Discussion

Cholangiojejunostomy is the most widely used surgical procedure for biliary stricture caused by various abnormalities. However, postoperative complications of this surgery are gradually highlighted with its extensive clinical application, especially complications of reflux cholangitis, anastomotic stoma stricture and intrahepatic lithiasis [5, 16, 17]. Severe grasp of surgical indications during the first operation, reasonable operative time, different anastomotic techniques and lack of full understanding of anastomotic techniques may result in postoperative anastomotic stoma stricture and intrahepatic lithiasis, which need further surgery for radical treatment. When second cholangiojejunostomy is performed for the patients, especially for those with traumatic biliary stricture, tissues around the portal hepatis are severely adhered and the anatomic level is unclear. Therefore, there may occur more complications after second cholangiojejunostomy [18, 19]. Chapman et al. have showed that failure rate of second cholangiojejunostomy in patients with biliary injuries was approximately 50.0%, and postoperative complications may also occur in patients with non-traumatic biliary stricture [10]. In this present study, a total of 61 patients underwent revisional Roux-en-Y cholangiojejunostomy for the second time due to biliary stricture, among which 21 still needed hepatectomy during the second surgery. In addition, Clavien-Dindo III and higher complications occurred in ten patients, whose incidence rate was 16.4% (10/61). During follow-up, three patients died and anastomotic stoma stricture was found in four patients, indicating that incidence rate of postoperative complications after second cholangiojejunostomy was relatively high. Therefore, preventing or properly treating postoperative complications of anastomotic stoma stricture and intrahepatic calculus after cholangiojejunostomy may improve surgical treatment efficiency. Through analyzing clinical data of 61 patients undergoing cholangiojejunostomy for the second time, this study demonstrated that first preoperative bilirubin level, short-term complication after first surgery and abnormalities during the second surgery were independent risk factors affecting patients' overall survival, suggesting that first surgery was the key to second or multiple cholangiojejunostomy that surgeons should take seriously in clinical work.

In this current study, first preoperative bilirubin level was one of the factors that may influence long-term surgical efficiency after cholangiojejunostomy. So far, there has been no consensus on whether drainage should be performed for patients with obstructive jaundice before cholangiojejunostomy. However, drainage should be performed before operation in the following situations: (i) Serum total bilirubin (TBIL) level is more than 250 µmol/L. (ii) Indocynine Green Rate 15 (ICGR15) is lower than 15.0%. (iii) Liver function was relatively poor. (iv) Patients are presented with clinical manifestation of cholangitis. (v) Patients are more than 70 years old. (vi) Patients are accompanied with basic diseases [20–24]. In our study, first preoperative total bilirubin level was more than 200 µmol/L in nine patients and there were varying degrees of short- and long-term postoperative complications in these patients, indicating that it was crucial to treat jaundice through biliary drainage when patient's preoperative bilirubin level was high. Additionally, short-term complications after the first cholangiojejunostomy were another influencing factor of patients' overall survival state. There occurred biliary fistula in six patients and intestinal obstruction in one patient. From the survival curve, it may be found that biliary fistula had significant postoperative effects on patients' survival and lasted for relatively longer time.

Abnormal findings during the second surgery were also of great significance after choledochojejunostomy. Among the patients, anastomotic stoma stricture was found in 42 patients with the highest incidence (68.9%). Compared with the patients with non-traumatic biliary stricture, patients with traumatic biliary stricture were more likely to have anastomotic stoma stricture. However, shorting of biliary output loop occurred in more patients with non-traumatic biliary stricture than patients with traumatic biliary stricture. In patients with traumatic biliary stricture, right posterior lobe biliary duct was end to side anastomosed with the jejunum in one patient, but the right anterior biliary duct and left hepatic duct were not anastomosed. In patients with non-traumatic biliary stricture, lower right posterior lobe biliary duct was not anastomosed in one patient during the first surgery, and cholangiojejunostomy was performed for the second time in another patient due to leaving some stones in the biliary duct of right posterior lobe during the first surgery. According to the survival analysis, it may be concluded that problems related to residual lesions occurred in the early stage after cholangiojejunostomy.

Anastomotic stoma stricture and shortening of biliary output loop occurred relatively later in patients. Most patients with traumatic biliary stricture had anastomotic stoma stricture before shortening of biliary output loop. Thus, second cholangiojejunostomy was performed for these patients. It was inevitable that there may appear biliary stricture after latrogenic biliary duct injuries, which often occurred two years after biliary repair. However, despite its rarity, there were still biliary related complications ten years after biliary repair. Stilling et al. have showed that the time of anastomotic stoma stricture after iatrogenic biliary duct injury repair ranged from 2 to 141 months [25]. Relatively, the average follow-up time for non-stricture patients was 102 months. Compared with patients with non-traumatic biliary stricture, cholangitis occurred earlier in patients with traumatic biliary stricture, and anastomotic stoma stricture occurred in more patients. However, biliary output loop related complications were more common in patients with non-traumatic stricture. For instance, shortening of biliary output loop was found in eight patients, calculous obstruction of biliary output loop in one patient, adhesive ileus of biliary output loop in one patient and internal hernia of biliary output loop in one patient. Relatively, shortening of biliary output loop and adhesive ileus of biliary output loop respectively occurred in three and two patients with traumatic biliary stricture. Collectively, combined with the above results, it was suggested that optimal biliary output loop used for cholangiojejunostomy was 40 ~ 60 cm. Too long biliary output loop used for drainage may be twisted, folded and obstructed due to adhesion, thus resulting in poor biliary drainage and increasing the chance of cholestasis and intestinal bacterial reproduction. In comparison, too short biliary output loop used for drainage may cause reflux easily [26, 27]. During follow-up, it was found that there was disuse atrophy of jejunum due to too short biliary output loop in some patients, whose length was less than 30 cm and even 20 cm, thus causing further narrow of the intestinal cavity.

Due to the retrospective characteristics of this study, there may exist some bias. Moreover, due to the small sample size, prospective studies are expected to be performed to further summarize the potential risk factors of cholangiojejunostomy in the patients complicated with biliary stricture. Despite the above limitations, this current study may provide valuable references for medical staff to realize and pay more attention to the possible postoperative complications of cholangiojejunostomy in their clinical work.

## Conclusion

In summary, postoperative complications are common clinical phenomenon in patients with benign biliary stricture after cholangiojejunostomy. Therefore, in order to minimize incidence of postoperative complications, it is necessary for surgeons to fully evaluate optimal operative time and procedures through preoperative imaging and combining with patients' overall health state. Moreover, standardized operation procedures may improve long-term surgical efficiency and patients' overall survival.

## Data Availability

The datasets used/analyzed during the current study are within the manuscript and more data could be available from the corresponding author on reasonable request.

## Abbreviations

CT: Enhanced computed tomography
MRI: Magnetic resonance imaging
PTBD: Percutaneous transhepatic biliary drainage
AST: Aspartate aminotransferase
ALT: Alanine aminotransferase
WBC: White blood cell
TBIL: Total bilirubin
ICGR15: Indocynine Green Rate 15
